# Laparoscopic management of cholecystocolonic fistula: A case report and a brief literature review

**DOI:** 10.1016/j.ijscr.2020.02.052

**Published:** 2020-02-28

**Authors:** G. Bonventre, G. Di Buono, S. Buscemi, G. Romano, A. Agrusa

**Affiliations:** Department of Surgical, Oncologic and Stomatological Disciplines (Di.Chir.On.S.), University of Palermo, Italy

**Keywords:** Cholecystocolonic fistula, Laparoscopy, Complicated cholelithiasis

## Abstract

**Introduction:**

Cholecystoenteric fistula is a rare and late complication of cholelithiasis. The clinical presentation is mostly chronic and is not distinguishable from the dyspeptic symptoms of non-complicated cholelithiasis. For this reason, the preoperative diagnosis is difficult and uncertain, and it is often made up primarily intraoperatively and incidentally during cholecystectomy. In this article, we report a case of cholecystocolonic fistula management by laparoscopic approach.

**Presentation of case:**

We studied a 64 years old male patient with fever of an unknown origin for two months and abdominal pain. He underwent a contrast enhanced CT abdominal scan that showed a sclerotic gallbladder with a disorganized fluid collection. The colonoscopy identified a cholecystocolonic fistula with hepatic flexure. A laparoscopic cholecystectomy was performed to repair the colonic wall with intra-corporeal sutures.

**Discussion:**

Thanks to the advancements in CT scan’s resolution and the application of endoscopic technology such as ERCP or colonscopy, preoperative diagnosis of cholecystoenteric fistula has been greatly improved. In addition, cholecystoenteric fistula has been successfully managed laparoscopically with laparoscopic cholecystectomy and closure of the fistula tract.

**Conclusion:**

The laparoscopic management of cholecystoeneteric fistula is a feasible and safe procedure but the operative strategy should be individualized on diagnosis, patient characteristics, availability of resources and experience of surgical team.

## Highlights

• Cholecystoenteric fistula is a rare and late complication of cholelithiasis.
• We report a case of cholecysto-colonic fistula with hepatic flexure management by laparoscopic approach, a 64 years old male patients with fever of an unknown origins for two months and abdominal pain.
• We performed laparoscopic cholecystectomy and repaired colonic wall with intra-corporeal sutures.
• The laparoscopic management of cholecystoeneteric fistula is a feasible and safe procedure but the operative strategy should be individualized.

## 1 Introduction

Cholecystoenteric fistula (CEF) is a rare and late complication of cholelithiasis that is defined as a spontaneous connection with bile flow between an inflamed gallbladder and one or more adjacent structures. Its incidence has been reported to occur in 3–5% of patients with cholelithiasis and in 0.15–4.8% of all patients who undergo surgeries on the biliary tract [[1], [2], [3], [4]]. The clinical presentation is mostly chronic and it is not distinguishable from the dyspeptic symptoms of non-complicated cholelithiasis such as abdominal weight and distention, eructation, nausea, pain in the right upper quadrant of the abdomen or back pain. For this reason, the preoperative diagnosis is difficult and uncertain and it is often is made up primarily intraoperatively and incidentally during cholecystectomy (0.5%) or by preoperatory endoscopic retrograde cholangiopancreatography (ERCP). In our case report, the diagnosis was made up by a preoperative colonoscopy. The rarity of this condition and the difficulties on its diagnosis justify the small number of articles in literature and, at the same time, the surgical approach is still in doubt. The “classic” treatment for this condition used to be open cholecystectomy and closure of the fistula eventually with its excision but recently, with the enhancements in laparoscopic surgery, several case reports described the potential use of the laparoscopic and robotic approach for this rare complication [2,5,6]. In this case report we evaluate the safety and risk of complications when the conservative laparoscopic approach is applied in patients with CEF in line with the SCARE criteria [7].

## 2 Presentation of case

**Fig. 1:**
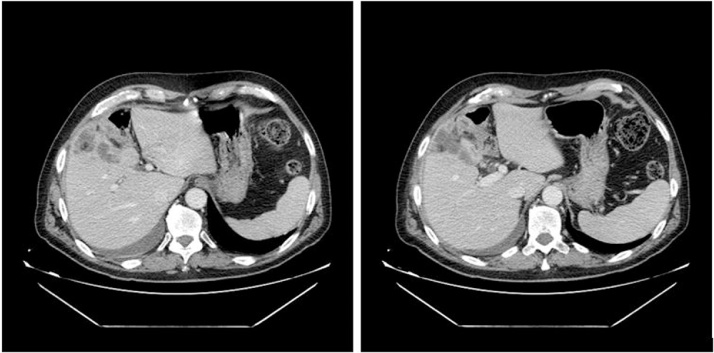
Abdominal CT scan shows a sclerotic gallbladder and right colonic flexure with walls harmonically thickened, with edema of the submucosa such as an inflammatory disease.

We studied a 64 years old male patient with fever of an unknown origin for two months and abdominal pain. He underwent a contrast enhanced CT abdominal scan that showed a sclerotic gallbladder with a disorganized fluid collection (6 × 1.7 cm), not surrounded by a clear wall and that presented no changes after the administration of intravenous contrast. Moreover, the right colonic flexure was thickened over 5 cms with edema of the submucosa indicating inflammation (Fig. 1). The colonoscopy identified a cholecysto-colonic fistula (CCF) with hepatic flexure. We decided for a laparoscopic approach at the CCF. Pneumoperitoneum by trans-umbilical incision was then performed with open technique and placed three other ports in left ipocondrio, in epigastric region and in right flank [[8], [9], [10]] respectively. In our procedures we used Veress needle for other laparoscopic procedures [[11], [12], [13]]. Through laparoscopic exploration, we found that the colonic hepatic flexure presented thickened walls in contact with the gallbladder and evidence of a clear CCF originated from the gallbladder’s body with remnant stones inside. A laparoscopic cholecystectomy was performed followed by repair of the colonic defect with intra-corporeal nonabsorbable sutures. At the end of the procedure, we decided to perform an intraoperative colonoscopy: during which maneuvers the careful observation did not demonstrate any problem with the colon. We performed umbical closure with detached sutures in order to avoid incisional hernia [14]. Postoperative course was uneventful with oral intake on POD 1. The patient was discharged on the 7th POD.

## 3 Discussion

Laparoscopic cholecystectomy (LC) is the most commonly performed surgical treatment for gallbladder disease. Amongst the rare and late complications of cholecystolithiasis it is possible to include cholecystoenteric fistula (CEF), defined as a spontaneous tract between an inflamed gallbladder and one or more adjacent gastrointestinal tract structures. According to the literature, the incidence of CEF occur in 3–5% of patients with cholelithiasis and in 0.15–4.8% of all patients who undergo surgeries on the biliary tract [1,2]. Cholecystoduodenal fistula (CDF) is the most frequently encountered type of CEF, comprising 75%–80% of all fistulas, followed by cholecystocolonic fistula (CCF) [13]. Because of the nonspecific symptoms of CEF compared with cholecystitis, the preoperative diagnosis of CEF is very difficult. Historically, CEF was always an incidental finding during surgical procedures and it was considered a contraindication for LC at the beginning of the laparoscopic era. With the improvement of CT scan resolution and the application of endoscopic technology such as ERCP or colonoscopy, preoperative diagnosis of CEF has been greatly improved [15]. In addition, CEF has been successfully managed laparoscopically in several cases and in high-volume centers. The main treatment for CEF is laparoscopic cholecystectomy and closure of the fistula tract. An alternative to intracorporeal or extracorporeal sutures for fistula closure is the use of endoloop or stapler [16]. In this case, the complication that can occur is the loss of the traction on the communicating organ with the subsequent retraction of the fistula stump outside the laparoscopic field of view. During this kind of surgery, also in high-volume centers, the rate of conversion is still very high due to the technical difficulties in intracorporeal suturing, in reducing inflammatory adhesions and in the complexity in the control of bleeding [2,[16], [17], [18]].

## 4 Conclusion

In this case report we show the opportunity of the laparoscopic approach for CCF. The procedure is feasible and the choice of the strategy to be employed should be individualized based on diagnosis, patient characteristics, availability of resources and experience of surgical team.

## Funding

None.

## Ethical approval

Ethical Approval was not necessary for this study. We obtained written patient consent to publication.

## Consent

We obtained written patient consent to publication.

## Author contribution

Giulia Bonventre: study design, data collections, data analisys and writing.

Giuseppe Di Buono: study design, data collections, data analisys and writing.

Salvatore Buscemi: study design, data collections, data analisys and writing.

Giorgio Romano: study design, data collections, data analisys and writing.

Antonino Agrusa: study design, data collections, data analisys and writing.

## Registration of research studies

Not necessary for this case report.

## Guarantor

Agrusa Antonino.

Romano Giorgio.

## Provenance and peer review

Not commissioned, externally peer-reviewed.

## Declaration of Competing Interest

Giulia Bonventre and others co-authors have no conflict of interest.
